# Free Allophonic Variation in Native and Second Language Spoken Word Recognition: The Case of the German Rhotic

**DOI:** 10.3389/fpsyg.2021.711230

**Published:** 2021-11-17

**Authors:** Miquel Llompart, Nikola Anna Eger, Eva Reinisch

**Affiliations:** ^1^Chair of Language and Cognition, Department of English and American Studies, Friedrich Alexander University Erlangen–Nuremberg, Erlangen, Germany; ^2^Institute of Phonetics and Speech Processing, Ludwig Maximilians University Munich, Munich, Germany; ^3^Acoustics Research Institute, Austrian Academy of Sciences, Vienna, Austria

**Keywords:** allophonic variation, phonetic variants, spoken-word recognition, visual-world eye-tracking, speech perception, rhotics, German, second language

## Abstract

The question of how listeners deal with different phonetic variant forms for the same words in perception has sparked great interest over the past few decades, especially with regard to lenited and regional forms. However, the perception of free variant forms of allophones within the same syllable position remains surprisingly understudied. Because of this, in the present study, we investigate how free allophonic variation in the realization of the German rhotic (/r/) impacts spoken word recognition for native German listeners and two groups of non-native listeners (French and Italian learners of German). By means of a visual-world eye-tracking task, we tested the recognition of spoken German words starting with /r/ when the rhotic was produced either as the more canonical variant, the uvular fricative [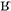] which is considered the German standard, or as an alveolar trill [r], a common realization in the south of Germany. Results showed that German listeners were more efficient at recognizing /r/-initial words when these were produced with the uvular fricative than with the alveolar trill. French listeners did not differ from German listeners in that respect, but Italian listeners showed exactly the opposite pattern: they showed an advantage when words were produced with the alveolar trill. These findings suggest that, for native listeners, the canonicity of the variant form is an important determiner of ease of recognition, even in the absence of orthographic or perceptual motivations for the primacy of canonical variants for this particular example of variation. For non-native listeners, by contrast, results are better explained by the match of the different allophones to the canonical realization of /r/ in their native language than by the status or frequency of the allophones in the non-native language itself.

## Introduction

Variation in language is ubiquitous. To begin with, languages differ in their phonological inventories, each of them combining a different set of phonological units to express word meanings. In addition to this, even within each language, phonological systems tend to show substantial variation at the phonetic level, meaning that the same phonological units can surface as greatly diverging phonetic forms. Some of this variation comes from speaker-specific sources, like the inherent physiological differences found between speakers (e.g., Hillenbrand et al., 1995; Deterding, 1997), and the effects that non-native accents can have on the pronunciation of particular speakers (e.g., Bohn and Flege, 1992; Eger et al., 2019; Llompart and Reinisch, 2019a), to name but two examples. However, there are also cases in which languages allow for multiple pronunciation variants for the same phonological units. This is what is commonly known as allophonic variation.

In some cases, allophonic variation may be fully constrained by the phonological context in which a given segment appears. For example, English /l/ can be realized as the alveolar lateral [l], which is known as “clear /l/” or as the velarized allophone [ɫ], frequently referred to as “dark /l/” (e.g., Jones, 1960; Roach, 1991; Hayes, 2009). The two allophones of English /l/ are in complementary distribution in that where one allophone is a suitable realization of /l/, the other is not. In short, /l/ is produced as [l] in onset position (e.g., *light*, *play*), and as [ɫ] in coda position (*ball*, *help*, but see also Turton, 2017). There are other instances of allophonic variation, however, which are not positionally constrained. Firstly, there are allophones that occur in the same phonological context and syllable position but whose usage is related to register, style and speech rate. They arise as alternations between full forms, also often referred to as citation or canonical forms, and one or more reduced forms. This includes allophonic variation stemming from phonological lenition processes like, for example, /t/ and /d/ tapping (Herd et al., 2010), nasal flapping (Patterson and Connine, 2001), or word final /t/ and /d/ glottalization (Sumner and Samuel, 2005; Seyfarth and Garellek, 2015) in American English. Secondly, a more extreme case of within-language allophonic variation is ‘‘free allophonic variation,’’ that is, when the different allophones can occur in free variation within the same context and syllable position without their use being constrained by register or speech rate. In those cases, the occurrence of the different allophones is only determined by idiosyncratic individual and regional variation. A case in point here is the realization of the German rhotic phoneme /r/,^1^ which is the focus of the present study.

The class of rhotics is exceptional in that, functionally, many languages share a phoneme /r/, yet the typical articulation of said phoneme can vary vastly in terms of place and/or manner of articulation (Lindau, 1985; Ladefoged and Maddieson, 1996). For instance, the prevalent variant of the rhotic in English is an alveolar or retroflex approximant (e.g., Delattre and Freeman, 1968; Lindau, 1985) while in Italian the rhotic phoneme is mostly realized as an alveolar tap or trill (Kaland et al., 2019), and in French as a uvular fricative (e.g., Rose and Wauquier-Gravelines, 2007; Boyce et al., 2016). Furthermore, evidence of variation in how /r/ is realized can often be found even within a single language, as for instance in German. In German, /r/ in syllable onset position can be realized as allophones that are radically different in terms of articulation and acoustics (e.g., Wiese, 1996, 2003; Schiller, 1998). In particular, among the different allophones of the German rhotic in onset position, the most prominent ones are the alveolar trill [r], the uvular trill [R], and the uvular fricative [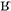] (Wiese, 1996). In the present study we investigate the consequences of this variation on the recognition of German words containing /r/ in word-initial position by native speakers of German, as well as two groups of second language (L2) learners of German: native speakers of Italian and French. We focus on the phonetically most distinct allophones [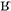] and [r], which share neither place nor manner of articulation.

### Allophonic Variation in Native Spoken Word Recognition

Free allophonic variation has different consequences for speech production and perception. Whereas in speech production this variation may afford speakers a certain amount of freedom in which variant form they use, in perception all possible variant forms have to be recognized as referring to a common phonological category. For example, in the case of German /r/, phonetically different forms such as [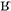
o:zə], [Ro:zə], and [ro:zə] have to be recognized as the German word *Rose* “rose” and crucially distinguished from other words such as, for instance, [ho:ze] *Hose* “pants.” The issue of how listeners recognize different phonetic variant forms has sparked great interest over the past few decades with regard to lenited and regional forms (Deelman and Connine, 2001; LoCasto and Connine, 2002; McLennan et al., 2003; Sumner and Samuel, 2005, 2009; Ranbom and Connine, 2007; Connine et al., 2008; Pitt, 2009; Bürki et al., 2010, 2011; Sumner, 2011; Bürki and Frauenfelder, 2012; Sumner and Kataoka, 2013; King and Sumner, 2014; Sumner et al., 2014; Llompart and Simonet, 2018). Despite this, to our knowledge, instances of free allophonic variation like that for German /r/ remain surprisingly understudied.

In fact, most research assessing the processing of allophonic variation in spoken word recognition to date has focused on variation contrasting full (i.e., citation) vs. reduced forms (e.g., *center* as ce[nt]er vs. ce[\~textfishhookr]er). Findings from this body of literature suggest that the most prominent factors determining listeners’ success at recognizing words with more than one pronunciation variant are the frequency with which each variant is used (e.g., Bürki et al., 2010) and the canonicity of each of the variants in the language (e.g., Ranbom and Connine, 2007; Sumner et al., 2014). Hence, words that are known to be more frequently produced and perceived with a given variant are usually recognized more easily when presented as containing that most frequent variant (Connine, 2004; Ranbom and Connine, 2007; Connine et al., 2008; Bürki et al., 2010; Llompart and Simonet, 2018). In spite of this, it has also been shown that, under some circumstances, native listeners are better and faster at recognizing words if they are presented with a variant form that is produced less frequently in everyday speech. This is the case when the less frequent form is considered canonical (e.g., center as ce[nt]er; LoCasto and Connine, 2002; Sumner and Samuel, 2005; Ranbom and Connine, 2007; Pitt, 2009).

Interestingly, parallel findings regarding canonicity have also been reported in relation to phonetic variation due to dialectal differences (Sumner and Samuel, 2009; Sumner et al., 2014; Sumner, 2015; Llompart and Simonet, 2018). Pronunciation variants of dialects or regiolects that are considered more canonical because of their social dominance have been found to be recognized with at least as much ease as variants associated to less canonical variants, even if the listeners are exposed to the regional variants more frequently. For example, Sumner and Samuel report that native New York City (NYC) residents who produced /r/-final words (e.g., *baker*) without r-coloring on the final vowel (bak[ə]) showed comparable priming in a cross-modal form priming task when primes corresponded to the variant associated to their own variety, and the more canonical, r-colored variant of General American (GA; bak[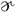]). Speakers of GA residing in NYC, by contrast, were only primed by their own r-colored variant, which at the same time is considered the standard. In consequence, the converging results across different types of variation have led to hypothesizing that both citation forms (e.g., ce[nt]er) and variants from dominant language varieties are more accessible to listeners because of their prototypical status (i.e., the *memory inequality* hypothesis in Sumner et al., 2014). However, the advantage for canonical forms has also led to the question as to what extent this advantage may relate to orthography (e.g., Bürki et al., 2018; Viebahn et al., 2018; Charoy and Samuel, 2020), as canonical variants tend to match the orthographic representation better than other variants.

In the present study we investigate how efficiently listeners recognize words starting with rhotic allophones. Specifically, we investigate the recognition of German words in which the rhotic is produced either as an alveolar trill ([r]) or a uvular fricative ([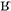]). The first research question we ask is whether native speakers of German will be faster at recognizing German /r/-initial words when the rhotic is produced as [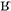] than when it is produced as [r]. Both [r] and [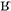] are encountered in Southern Germany, where the present study took place. However, the uvular fricative is generally ascribed to Standard German as the canonical realization of the rhotic and is the one that is prevalent in national media. The alveolar trill, by contrast, is common in Southern varieties of German and is also used in Bavarian dialects. Therefore, building on the research outlined above (e.g., Sumner et al., 2014), the canonical status of [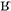] could in principle result in this variant being recognized more efficiently by native speakers of German, even if listeners living in the German South should also be familiar with the alveolar trill.

Assessing the role of German rhotic allophones in word recognition is particularly interesting because this differs from the phenomena examined in previous studies in critical ways and can thus provide additional insights on the factors constraining the recognition of multiple variant forms. First of all, we chose to investigate the two German rhotic allophones that maximally differ in their phonetic forms, that is, the alveolar trill [r] and the uvular fricative [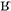]. Note that these two allophones do not share any phonetic properties as they differ in place and manner of articulation. This is critically in contrast to previous studies on, for instance, nasal flapping in American English, where some acoustic similarity between variant forms is expected due to a match in place of articulation. Secondly, and relatedly, the two rhotic allophones considered do not instantiate an opposition between a full variant (i.e., citation form) and one or more reduced variants. Thirdly, regardless of the allophone of the rhotic that is used, the associated spelling should always be <*r*>, without any *a priori* difference in match between phonetic form and orthography. This is again in contrast to previous research. Finally, testing the effects of this particular type of allophonic variation is of interest because articulatory and acoustic properties of the variants in question do not go hand in hand with canonicity: Whereas the uvular fricative [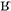] is the more canonical and frequent variant of these two, the alveolar trill [r] could be considered the more perceptually salient variant based on its articulatory and acoustic properties.

The alveolar trill is an articulatorily complex sound that needs to reach very precise articulatory targets (Solé, 1999, 2002). Alveolar trills are acquired rather late by L1 speakers (Jiménez, 1987; Vihman, 1996; Boyce et al., 2016) and learning to produce them is often associated with serious difficulties in L2 learning (Major, 1986; Face, 2006). However, in speech perception, voiced trills are thought to be quite salient (Steriade, 1999; Solé, 2002; Bradley, 2006; Colantoni and Steele, 2007; Sebregts, 2015) because their trilled manner of articulation results in “a clearly modulated signal, distinct from other speech segments” (Solé, 2002, p. 682), which should aid their perceptual identification. Crucially, in addition to their perceptual salience, alveolar trills are also phonetically quite distinct from other German phones. No other phone of German is produced with a similar manner of articulation to the alveolar trill other than the uvular trill, which is another allophone of the rhotic. By contrast, the uvular fricative is much less distinct, as German has many fricatives, crucially including other “back” fricatives like [x] (as an allophone of /ç/) and /h/ which are acoustically similar to [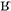]. Hence, considering that previous studies suggest that contrasts in manner of articulation are discriminated more accurately than contrasts in place or voicing (Martin and Peperkamp, 2015, 2017), it could be predicted that, in terms of bottom-up perception of the speech signal, [r] should be at an advantage in recognition over [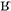]. This may thus interfere with the advantage for [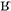] that could be expected given that it is considered the standard, canonical form. The present results will thus also speak to this issue.

### Allophonic Variation in L2 Spoken Word Recognition

The need to recognize multiple phonetic forms as instances of the German rhotic does not only apply to native speakers. Learners of German as a second language (L2) are also faced with this challenge. They need to learn that, in order to be able to efficiently recognize L2 words (e.g., *Rose*), very different allophones have to be associated with the same phonological category. Therefore, the second research question of this study is whether L2 learners of German living in Germany will differ from native speakers in how efficiently they recognize words with the two allophones of German /r/ (i.e., [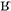] and [r]) and, if so, to what extent these differences may be related to the characteristics of the rhotics in their L1s. In order to answer this question, a group of French learners of German and a group of Italian learners of German were tested in addition to native speakers of German. While much research has been devoted to the effects of phonetic variation in L1 speech perception, little is known about the consequences of this variation in second, later-learned languages. This is partly because, in the literature on L2 learning, the focus is typically on phones that are similar in the native and non-native language and specifically on cases where multiple L2 phones are phonetically close to the same L1 category (e.g., English /ε/ and /æ/ for German learners of English; Bohn and Flege, 1990; Llompart and Reinisch, 2019b). Therefore, the scenarios that are commonly of interest in L2 learning research are practically the opposite to the situation that L2 learners face with the allophones of German /r/.

Given the large articulatory and acoustic differences between [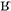] and [r], assessing the effects of allophonic variation for German /r/ on L2 spoken word recognition is especially relevant in cases where these allophones differ in how close a phonetic match they are to the canonical realization of the rhotic in the learners’ L1. This is precisely the case for French and Italian learners of German. While variation in the articulation of /r/ can be found in both languages, especially as far as regional dialects and varieties of bilingual communities are concerned (see Canepari, 1979, 1990; Bertinetto and Loporcaro, 2005; Sankoff and Blondeau, 2007; Webb, 2009; Romano, 2013), the canonical variant of the French rhotic in mainland France is the uvular fricative [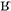] (Rose and Wauquier-Gravelines, 2007), whereas in Italian /r/ is canonically realized as an alveolar tap or trill (Kaland et al., 2019). For the purposes of the present study, these canonical forms will serve as the reference for comparison with the native German listeners.

Most prominent models of L2 phonology acquisition (e.g., Flege, 1995; Best and Tyler, 2007; Van Leussen and Escudero, 2015; Flege and Bohn, 2021) generally assume that L2 phonological categories that are phonetically very similar (i.e., close to identical) to L1 phonemes are easy to incorporate into the L2 phonological inventory and to use in L2 perception. Because of this, one could hypothesize that L2 spoken word recognition for words with a rhotic should be aided when the rhotic is realized as the allophone that matches the canonical form of /r/ in the L1 of the learners. French learners would thus be expected to be faster at recognizing German words when /r/ is realized as [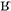] and Italian learners when it is realized as [r]. Partial support for this hypothesis comes from a recent study by Reinisch et al. (2020), which showed that Spanish learners of German were more accurate in a word identification task with German /r/-/h/ minimal pairs (e.g., *Rose*-*Hose* “rose-pants”) when /r/ was produced with a trilled manner of articulation, like in their L1, than when it was produced as a uvular fricative. In spite of this, it is also possible that recognition patterns for L2 learners are not as clear-cut as this given that, by virtue of their linguistic immersion in Southern Germany, L2 learners are also expected to be susceptible to the effects of variant frequency and canonicity described above in relation to native German speakers. Similarly, the discussion on the perceptual salience of the two allophones also applies to the potential outcomes for non-native speakers.

### The Present Study: Summary and Predictions

In the present study, we tested the recognition of German words starting with the rhotic phoneme produced either as [r] or [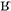] by means of a visual-world eye-tracking task (e.g., Llompart and Reinisch, 2017; Eger et al., 2019). In this task, participants are asked to listen to spoken sentences while viewing visual displays to find potential referents. Throughout the task participants’ eye gaze patterns are captured. Since gaze patterns are closely related and time-locked to the acoustic input that listeners receive (Allopenna et al., 1998), this allows for the on-line examination of spoken-word recognition over time, and here specifically of the impact of the allophone on the efficiency of word recognition (as measured through fixations on the target visual referent). As discussed above, three groups of participants were tested: a group of native speakers of German and two groups of L2 learners of German with French or Italian as L1. At the time of testing, all participants were living in Southern Germany and were hence familiar with the uvular fricative as the German standard as well as the alveolar trill as part of local regional varieties. Summarizing, our predictions are the following:

• P1: Native speakers of German will be better at recognizing the target words when the stimuli are produced with [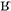].

We expect an advantage for [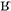] due to its status as the canonical/standard variant and possibly its overall higher frequency due to national media. Nonetheless, the higher perceptual saliency of [r], together with the fact that [r] is also common in Southern Germany could counteract that tendency.

• P2: L2 learners will be better at recognizing the target words whenever the allophone of /r/ matches the canonical realization of the rhotic in their native language.

We expect French learners of German to show an advantage for [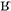] (similarly to L1 German speakers) and Italian learners of German to show an advantage for [r]. While frequency and canonicity in the L2 and perceptual salience may compete with this L1 preference, their influence is expected to be weaker for L2 than for native listeners because of the attracting force of the learners’ L1 phonology.

## Materials and Methods

### Participants

In the present study, 24 native speakers of German, 21 native Italian and 25 native French learners of German took part for a small monetary compensation. The criteria for recruitment for all groups were monolingual upbringing speaking German, Italian, or French, in Germany, Italy, or France, respectively. The age at testing was to be between 18 and 45 years, and no diagnosed speech or hearing impediments or dyslexia should be reported. For the two learner groups an additional requirement was that, according to self-report, their level of German was between B1 and B2 according to the Common European Framework of Reference for Languages (CEFR). This was to ensure that they would be able to understand the speech material used in the task but prevent ceiling performance that might obscure any potential differences between learner groups.

As for the final set of participants, the native speakers of German were aged between 18 and 31, with a median age of 25 years. Fifteen of them were born and raised in the south of Germany, in Bavaria or Baden-Württemberg, while the rest grew up in other regions such as Hessen, North Rhine-Westphalia, and Brandenburg. Importantly, all participants had been living in Bavaria for at least 2 years and were hence familiar with the local language varieties. All had learned English at school and reported some knowledge of additional languages such as French, Italian, or Spanish (from school). Twenty-two of them were students at Ludwig Maximilians University Munich.

The Italian participants were aged between 22 and 43 with a median age of 29. They came from different regions of Italy, including Lombardia, Campania, and Emilia Romagna, as well as the islands of Sicily and Sardinia. Nine participants reported being able to speak a specific dialect of Italian.^2^ They reported having lived in Germany for about two and a half years on average, and had started learning German at a median age of 23 (the youngest starting age being 7 and the oldest 39). They all had learned English at school. In a self-assessment questionnaire devised after the experiment, participants rated their Italian accent in German on a scale from 1 (very weak) to 7 (very strong) at an average of 4.9. Additionally, they were asked to rate their skill level in German from 1 (very good) to 7 (very bad) in the categories, speaking, listening comprehension, writing, reading, and overall proficiency. The average response was 3.8 for speaking, 3.1 for listening comprehension, 4.0 for writing, 3.2 for reading, and 3.4 for overall German proficiency.

The French participants were aged between 19 and 44, with a median age of 27. They grew up in various regions of France (e.g., Aquitaine, Île-de-France, and Normandy) and reported having learned some English at school before they started learning German at a median age of 23 (the youngest starting age being 9, the oldest 29). Only two participants reported using a specific dialectal variant of French regularly (one from Alsace, one from the ‘‘south of France’’). At the time of testing, similarly to the Italian learners, they had spent an average of two and a half years in Germany. Their self-assessed French accent in German was 5.2 on average. Their average reported skill levels were 3.6 for speaking, 2.6 for listening comprehension, 3.9 for writing, 3.1 for reading, and 3.3 for overall German proficiency.^3^

### Material

The overall design of the experiment was inspired by Eger et al. (2019) on the perception of /h/ and /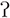/ by Italian learners of German. Forty /r/-initial German nouns of 1–3 syllable length were selected as targets such that they were picturable and likely known to intermediate learners of German. Sixty further words were selected as fillers. Fillers started with various other German phones, including some that were expected to be “easy” for our learners, and others that were known to be “difficult,” as for instance /h/. In this way, listeners were prompted to not focus on only one particular phonological category. For each of these 40 /r/-initial and 60 filler targets, a context sentence was generated such that the critical word occurred in sentence final position. For each target a semantic competitor and a distractor word was chosen, both of which were phonetically different from the target and from each other (i.e., no phonological overlap at word onset or offset). We refer to a word as semantic competitor if it also fit the context sentence and as distractor if it did not fit the context. For instance, for the sentence “Der Kellner kommt mit der” (English: the waiter brings [literally: comes with] the) the word triplet was “Rechnung/Suppe/Decke” (English: check, soup, and comforter) where target and competitor both fit the context (i.e., check and soup) but the distractor (comforter) did not. Except for the /r/-targets, no other instances of /r/ in syllable onset occurred in the sentences or other words. Note that /r/ in syllable coda was not possible to avoid, but since it is typically vocalized in Southern Germany (Wiese, 2003) – and was consistently vocalized by the speaker who recorded the stimuli – those instances should not interfere with the present design.

For all targets, competitors, and distractors, pictures were selected from a database in which the authors kept previously used images or *via* a Google image search. Pictures were in color and re-formatted to the same size of 400 × 300 pixel. Triplets of target, competitor, and distractor pictures were selected to be visually distinct to minimize competition due to visual resemblance (Huettig and McQueen, 2007).

Words within a triplet were roughly matched in log-frequency estimated from the SUBTLEX-DE corpus (Brysbaert et al., 2011), which takes into account spoken forms (mean /r/ targets: 2.34, competitors to /r/ targets: 2.38, distractors to /r/ targets: 2.35; mean filler targets: 2.33, competitors to filler targets: 2.21, distractors to filler targets: 2.2). In addition, relative to the context sentences, competitors were selected such that competitors to /r/ targets were slightly more probable given the context than the /r/ targets. This was to prevent participants from anticipating the target before it had been heard, which might have obscured any effects of the allophone of /r/ that had been produced. However, over the whole experiment, target, and competitor items were approximately equally likely. That is, the slight imbalance in probability between /r/-targets and their competitors was counteracted by making the target somewhat more likely in some of the filler trials. The fit of targets and competitors to the sentence contexts was confirmed in a written web-based pretest.

In the pretest, all sentences were presented in written form ending in the targets and competitors. Participants were asked to rate on a scale from 1 “very good fit” to 5 “does not fit at all” how well a given word fit the context sentence. The pretest was run as a web-based survey. Responses were obtained from 266 participants. In a few instances in which the fit of a given word and its competitor was rated as drastically different and thereby mismatched the overall pattern in a given word set, one of the words and pictures was replaced based on the authors’ opinion. Overall, the patterns described above were confirmed. An additional purpose of the pretest was to split the 40 /r/-initial words into two sets such that half of the words were presented with the uvular fricative allophone and half with the alveolar trill, while maximizing comparability of sets with regard to the fit to the context. This was done by sorting the words according to the difference between target and competitor fit to the context as assessed in the pretest and putting every second item into one or the other list (i.e., the tokens ranked 1, 3, 5, etc., went to one list, and the tokens ranked 2, 4, 6, etc., went to the other list).

### Recordings

Recordings were made in a sound-attenuated booth at the institute of Phonetics and Speech Processing at Ludwig Maximilians University Munich using a large-diaphragm condenser microphone (Neumann Microphone, type TLM 103) and SpeechRecorder software for sentence presentation and storage (Draxler and Jänsch, 2004). Recordings were made by a female native speaker of German who naturally produces a back fricative variant of /r/ but is able to produce an apico-alveolar trill. Filler sentences were recorded once and repeated only if upon the first recording an error or a hesitation occurred. Sentences with /r/-initial targets were recorded several times with the targets being produced with a uvular fricative as well as an alveolar trill. The speaker took care to match sentences in overall speech rate and intonation contour such as to facilitate the splicing procedure described below.

Recordings of /r/-targets were cut and spliced in Praat (version 6.0.36, Boersma and Weenink, 2009). In all recordings the beginning of the /r/ was assessed by combining auditory impression and visual information from the spectrogram and oscillogram. For each target word, a recording of the context sentence was selected that was spoken clearly and did not contain any hesitations or unusual prosody. In addition, a token of the target word with the fricative and a token with the trill were selected from different recordings and spliced onto the selected context sentence. If one of the targets happened not to fit the context sentence well (e.g., if clicks were audible at the splicing point) new tokens of the sentence or target were selected. Fillers were not spliced. For these items, whole sentences including the targets were selected according to the same criteria as the sentences for /r/-initial targets. For all items, the target onset was identified auditorily and by visual inspection of the spectrogram in order to lock target fixations in the analyses.

### Design

Each participant heard 100 sentences: All 60 filler trials and 40 sentences with /r/-initial targets, half with the uvular fricative and half with the alveolar trill. The split was determined as described with the pretest above. The set of targets with each allophone was counterbalanced across participants. That is, each participant heard all words once but was presented both allophones of /r/. Note that the design aimed at comparing fixations on the target in the different allophone conditions, not the preference of target over its competitor.

The order of trials was randomized separately for each participant. The three pictures per trial were presented in the four quadrants of the screen (leaving one quadrant empty) such that for each participant, target, and competitor appeared equally often in each position. This was to prevent any potential target bias based solely on the position where participants typically scan the screen starting at the top left position.

### Procedure

Participants received oral and written instructions in German. They were asked to listen to German sentences and click with the computer mouse on the visual item that best fit the sentence. If participants indicated that they had not understood the task they could ask questions and were provided with clarifications.

On each trial participants first saw a fixation cross in the middle of the screen for 700 ms. Then the three pictures appeared on the screen and the fixation cross was replaced by the mouse cursor. The start of the sound file was timed such that the target occurred 3000 ms into the trial, that is 2300 ms after the pictures appeared on the screen. To prevent participants from clicking the mouse before they heard the audio, the mouse cursor could be moved only from 500 ms before target onset. After participants clicked, a blank screen appeared for 700 ms before the next trial started. No feedback about the answers was given. Halfway through the experiment, that is, after 50 trials, participants could take a short self-paced break which they ended by clicking with the mouse.

Pictures were presented on a 19-inch screen approximately 60 cm from the participants’ head. Audio stimuli were presented over headphones at a comfortable listening level. The experiment was conducted running Psychopy2 (v.1.83.01; Peirce, 2007) and eye fixations were collected by means of an Eye Tribe portable eye-tracker (The Eye Tribe Aps, Copenhagen, Denmark) at a rate of 60 Hz. The eye-tracking part took approximately 15 min to complete.

After the experiment, participants were required to fill in a language background questionnaire which for the learners also included questions asking them to rate their proficiency in speaking, listening, reading, and writing in German as well as a self-assessment of their accent when speaking German, as described above in the Participants section. Additionally, they were provided a randomized list of the target words used in the experiment where they could indicate if a given word was unfamiliar to them or if they thought they had heard the word before but did not know its meaning. Those words were excluded from the analyses for the respective participants (see below).

## Analyses and Results

Data from two native German participants were excluded from analyses because they indicated that they grew up bilingually and so were data from two French and four Italian learners of German because of eye-tracker malfunctions. The final dataset that entered statistical analyses hence contained data from 22 native German speakers, 23 French learners of German, and 17 Italian learners of German. For the L2 learner data, trials that contained targets that learners classified as unknown words were additionally removed. This resulted in the exclusion of 182 trials (7.91% of the data) for the French group and 115 trials (6.76% of the data) for the Italian group. Finally, analyses on eye-fixation data were only conducted on trials in which participants clicked on the correct targets. A click was defined as correct when it was within the quadrant of the screen in which the picture corresponding to the target word was shown. Response accuracy was very high in all groups [German: 99.1% correct (SD = 9.5); French: 97.4% correct (SD = 15.9); and Italian: 97.7% correct (SD = 15.1)]. Discarding incorrect responses only led to the removal of 20 trials for the German group, 55 trials for the French group and 37 trials for the Italian group.

**FIGURE 1:**
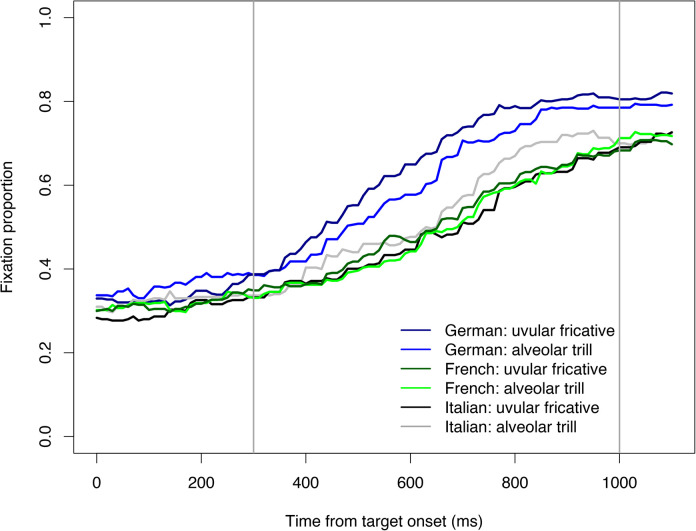
Proportion of target fixations over time to /r/-initial targets by allophone of /r/ (alveolar trill vs. uvular fricative) for each of the three listener groups. Zero indicates acoustic target onset. Lines for German listeners are in dark vs. light blue, for French listeners in dark/light green, and Italian listeners in black/gray. The darker colors refer to the uvular fricative allophones, the lighter lines to the alveolar trill. The gray vertical lines indicate the time window of analyses.

Statistical analyses were carried out similarly to Eger et al. (2019) on target fixation data, which was the logOdds-transformed proportion of fixations on the target picture over the time window from 300 to 1000 ms after target onset. The onset of the time window was set to 300 ms to account for the fact that L2 learners are typically slower than native speakers in responding to acoustic input, which is also reflected in eye-fixation data (e.g., Weber and Cutler, 2004). The offset of the time window was set to 1000 ms because this approximately corresponds to the point in time when target fixations stabilize for all groups (see Figure 1 below). Data corresponding to trials with /r/-targets in which a correct answer was provided were submitted to a linear mixed-effects model (lme4 package 1.1–26 in R version 4.0.3; Bates et al., 2015) with logOdds-transformed target fixations as dependent variable and Rhotic Allophone (fricative/trill), listeners’ L1 (German/French/Italian), and the interaction between these factors as predictors. Variables were factor coded with levels named as mentioned above. That is, the uvular fricative allophone of the rhotic and German as native language (i.e., L1 listeners) were mapped onto the intercept. The random-effects structure included random intercepts for Participants and Items. Adding random slopes for Rhotic Allophone over Participants and listeners’ L1 over Items did not improve the model’s fit as assessed through log-likelihood ratio tests. Therefore, no random slopes were included in the model. Significance of variables was assessed by means of Satterthwaite’s approximation for degrees of freedom using the lmerTest package (version 3.1-3; Kuznetsova et al., 2017).

Figure 1 shows target fixations over time for the three listener groups split by Rhotic Allophone. This figure depicts how, across all trials with /r/-initial targets, the proportion of fixations to the target referent increased over time (i.e., from target onset at 0 until 1100 ms after target onset) as a function of the allophone of /r/ that was produced for each group. Additionally, separate figures for each of the three participant groups reporting fixations on all visual referents (i.e., target, competitor, and distractor) are provided as Supplementary Material. The results of the statistical model are reported in Table 1.

**TABLE 1:** Results of the mixed-effects model on the effects of Rhotic Allophone and L1 on target fixations for /r/-targets with the reference levels L1 German and uvular fricative.

**Predictor**	***b***	***t***	***p***
Intercept	1.01	4.39	<0.0001
L1: French	−0.98	−3.24	=0.002
L1: Italian	−1.11	−3.38	=0.001
Rhotic allophone: trill	−0.26	−1.8	=0.072
L1: French × rhotic allophone: trill	0.22	1.08	0.28
L1: Italian × rhotic allophone: trill	0.61	2.72	=0.007

Results show that for the uvular fricative allophone of the German rhotic -- the level of Rhotic Allophone that had been mapped onto the intercept -- targets were fixated on more by native speakers of German than either of the two learner groups.^4^ This can be seen by the effects of L1 French and L1 Italian with a negative sign of the estimate. Moreover, this difference is also clearly visible in Figure 1 where the blue lines representing target fixations by native German listeners are substantially higher than the lines representing the learner groups’ fixations over the whole time window of interest. Crucially, the marginally significant effect of Rhotic Allophone: trill (again with a negative estimate) suggests that for native German listeners fewer target fixations were made if the target was produced with the alveolar trill than when it was produced with the uvular fricative. This is illustrated by the difference between the dark and light blue lines in Figure 1. The dark blue line corresponding to targets spoken with the fricative allophone is higher than the light blue line showing fixations for auditory targets with alveolar trills. The lack of a significant interaction between L1 French and Rhotic Allophone: trill further suggests that, with regard to differences in target recognition between the two rhotic allophones, French learners of German did not behave differently from the German native listeners. The presence of a significant interaction between L1 Italian and Rhotic Allophone: trill, in contrast, suggests that Italian learners did significantly differ from German listeners in their reaction to the different rhotic allophones.

In order to further explore this observation, a mathematically equivalent statistical model was fit, this time mapping Italian learners and the alveolar trill onto the intercept. Results are summarized in Table 2 and show that Italian learners of German fixated on the /r/-initial targets more when they were produced with [r] than when they were produced with [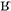]. This is indicated by the significant effect of Rhotic Allophone: fricative. In Figure 1 this can clearly be seen by comparing the gray and black lines. The gray line represents Italian learners’ target fixations for words produced with [r] and is higher than the black line, which depicts target fixations for [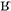]. The interaction Rhotic Allophone: fricative × L1 German shows the identical effect reported above. The interaction with L1 French just fails to reach significance and is likely contingent on the lack of difference between L1 French and native German speakers. Fixations for French L2 learners are shown in Figure 1 by means of the dark and light green lines, which are both found between the gray and black lines for the Italians. In sum, the native German listeners were more efficient at target recognition if the heard allophone was [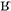], Italians showed the opposite pattern and French learners fell in between the two other groups.

**TABLE 2:** Results of the mixed-effects model on the effects of Rhotic Allophone and L1 on target fixations for /r/-targets with the reference levels L1 Italian and alveolar trill.

**Predictor**	***b***	***t***	***p***
Intercept	0.26	0.99	0.327
L1: German	0.49	1.51	0.134
L1: French	−0.26	−0.80	0.426
Rhotic allophone: fricative	−0.35	−2.04	=0.042
L1: German × rhotic allophone: fricative	0.61	2.72	=0.007
L1: French × rhotic allophone: fricative	0.39	1.71	=0.087

## Discussion

The present study set out to test how native speakers of German and two groups of L2 learners of German auditorily recognize German words starting with the phoneme /r/. Critically, the rhotic was produced as either of two radically different allophones, the uvular fricative [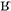] or the alveolar trill [r]. The results of a visual-world eye-tracking study showed that native German listeners fixated on referents to /r/-initial target words more when the rhotic was produced as a uvular fricative than when it was an alveolar trill. The two groups of L2 learners, which consisted of native French and Italian speakers residing in Southern Germany, were overall slower at fixating on the intended referents than the native listeners, just as expected. In addition, divergences in the effect of allophone were observed for the two groups in relation to native speakers: French listeners, whose L1 rhotic is canonically produced as a uvular fricative, did not significantly differ from native German listeners. Italian listeners, by contrast, showed the exact opposite pattern to native German listeners, fixating on the targets more when the rhotic was produced as an alveolar trill, which is the allophone that corresponds to the canonical realization of the rhotic in their L1.

### Canonical Advantage in L1 Spoken Word Recognition

The first and perhaps the most relevant finding of this study is that native speakers showed a recognition advantage for the allophone that is dominant at a national level and could be considered the canonical variant in Standard German. This is in principle not surprising, as our results parallel those of previous studies assessing the effects of phonetic variation for style- and register-conditioned allophonic variants opposing full and reduced forms (Deelman and Connine, 2001; LoCasto and Connine, 2002; Sumner and Samuel, 2005; Ranbom and Connine, 2007; Pitt, 2009; Bürki et al., 2011) and for dialectally diverging variant forms (Sumner and Samuel, 2009; Bürki et al., 2010; Llompart and Simonet, 2018). However, as already discussed in the Introduction, our study focuses on an instance of variation that differs in important ways from those examined before, which allows us to extend previous findings in two clear directions while overcoming some of the limitations faced in prior research.

In the first place, here we contrast the recognition of two allophones that could both be considered “full” variants in that none of them is a reduced (or even elided) form of the other (Mitterer et al., 2013; Sebregts, 2015; Ribbens-Klein, 2016). This stands in contrast to all studies assessing variation stemming from phonological processes like /t/ and /d/ tapping (e.g., McLennan et al., 2003), nasal flapping (e.g., Pitt, 2009), and schwa deletion (e.g., LoCasto and Connine, 2002), where the opposition between full and reduced form is the typical scenario. Studies on the recognition of dialectally conditioned variation have similarly focused on variants differing in the presence or absence of vowels (Bürki et al., 2010), particular vowel features (i.e., r-coloring; Sumner and Samuel, 2009), or spectral vowel reduction (Llompart and Simonet, 2018). Hence, the observed recognition benefit for the canonical variant of the German rhotic crucially indicates that canonicity effects can also arise for variants exclusively involving full albeit phonetically extremely dissimilar realizations of the same phonological category. This suggests that the advantage for canonical forms in previous studies is not only due to the fact that canonical variants tend to be longer, clearer, and perceptually more salient than their reduced counterparts. In fact, note that, for the allophones in the present study, it is the alveolar trill that was expected to be more salient than the canonical uvular fricative (Steriade, 1999; Solé, 2002; Bradley, 2006; Colantoni and Steele, 2007; Sebregts, 2015). Our results thus point toward the idea that, for native listeners who are not expected to have perceptual difficulties with their native phonological system, perceptual salience may not be a critical factor in determining how different pronunciation variants tap into lexical representations during spoken word recognition.

Secondly, for [r] and [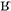] as allophones of German /r/, there is no difference in how well the two allophones correspond to the orthography of the target phonological category (i.e., <*r*>). This again contrasts with previous research, where both the full variants or citation forms in studies of reduction processes and the variants of the more canonical dialects in cross-dialectal word recognition had clearer associations with orthography. For example, for nasal flapping (e.g., Ranbom and Connine, 2007), the canonical variant consists of two segments, [n] and [t], which straightforwardly matches the orthographic string <*nt*>, whereas the nasal flap presents a mismatch in that it is just one segment and the orthographic <*t*> in <*nt*> does not have a phonetic counterpart when the flapped allophone is used. Similarly, in Sumner and Samuel (2009), where an advantage for the canonical GA form of -er final words was found over the form that is typical of the NYC dialect, the former contains r-coloring (e.g., bak[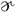]), which makes their association with orthographic representations like *baker* clearer than for the NYC r-dropping forms (e.g., bak[ə]).

This potential confound has brought about more recent research questioning to what extent the advantage for canonical forms may be driven by orthographic influences (Bürki et al., 2018; Viebahn et al., 2018; Charoy and Samuel, 2020). These studies, which have once again focused on reduction processes entailing full and reduced variants, and the subsequent asymmetric matches with orthographic representations, have led to somewhat mixed conclusions. On the one hand, building on their results in a novel word learning paradigm assessing /t/ and /d/ tapping and /nt/ flapping, Charoy and Samuel (2020) claim that orthography is indeed the driving force behind the canonical-form advantage. By contrast, Bürki et al. (2018) and Viebahn et al. (2018) assessed the recognition of variant forms for schwa deletion in French and argue for a less prominent role of orthography in spoken word recognition, especially in comparison to the effects of variant frequency and input variability during learning. The present results for native German speakers align more closely with the results of Bürki et al. (2018) and Viebahn et al. (2018), given that, in a situation in which orthography should *a priori* not bias variant recognition, we still find an advantage for the canonical allophone. This could therefore be interpreted to suggest that an advantage for canonical variants is tangible even when no case for it can be made based on orthographic considerations.

### L1 Influence on /r/-Word Recognition for L2 Learners

Our second research question was whether L2 learners of German living in Germany, who were bound to also be exposed to allophonic variation for German /r/, differed from native speakers in how efficiently they recognized words with the two German rhotic allophones. Specifically, we asked to what extent potential differences could be explained by the canonical realization of the rhotic in the learners’ L1s. We predicted a recognition advantage for the rhotic allophone that results in a closer match between L1 and L2. Indeed, we found that French learners of German, whose L1 has the uvular fricative as the standard realization of /r/ in France, were not significantly different from native German listeners, who fixated the targets more when the heard allophone was [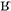] than when it was [r]. By contrast, Italian learners of German recognized words that were produced with the alveolar trill, the allophone that was expected to better match their L1, more efficiently than words with the uvular fricative (see Figure 1).

The results for the Italian learners of German suggest that these listeners indeed benefited from the match between the alveolar trill in German words and the typical realization of their native language. This aligns with the shared claim of most L2 phonology learning models (e.g., Flege, 1995; Best and Tyler, 2007; Van Leussen and Escudero, 2015; Flege and Bohn, 2021) that categories that are phonetically very similar, or even shared in L1 and L2, are easy to incorporate in the non-native phonological inventory and use in L2 perception. What is more, the present study shows that this relative ease in integrating L1 categories into L2 processing extends to spoken word recognition in tasks that require a higher degree of lexical involvement (Llompart, 2019, 2021; Llompart and Reinisch, 2019b) than the lower-level phonetic identification and discrimination tasks that are commonly used in research on L2 phonological acquisition (Bohn and Flege, 1990; Iverson and Evans, 2007; Rallo Fabra and Romero, 2012). Interestingly, the Italian learners as a group were aided by the alveolar trill despite their diverse regional origin and also in spite of the fact that the uvular fricative is thought to be the canonical variant of the rhotic in German. Although further research on the topic will need to confirm these impressions, the Italian learners’ results suggest that canonicity effects in a given language may be weaker for learners than for native speakers because of learners’ more unstable L2 phonological systems. In addition, canonicity effects of the target language may be overridden by the considerable influence that the native phonological system exerts on non-native listening. That is, the canonical form of the L1 may override effects of canonicity in the L2 when attending to the L2.

Finally, while the French learners of German did not differ from the native German listeners in terms of their use of the allophonic variants for target recognition, Figure 1 suggests that the advantage for [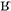] over [r] was larger for the native German listener group. While this needs to be interpreted with caution in the absence of a significant difference, following from the argument above, one could speculate that results for French learners are less clear because of their still-developing L2 phonological systems. In principle, both the higher canonicity of [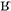] in German and the match between this allophone and the canonical realization of French /r/ motivate the prediction that [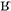]-targets should be more efficiently recognized than [r]-targets by these learners. However, as discussed in the Introduction, [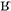] is at a disadvantage if one considers its place within the German phonological inventory, for the alveolar trill is not only perceptually more salient, but also more distinct from any other German phone in terms of articulation and acoustics. Hence, in on-line spoken word recognition, it is not unthinkable that French learners may have benefited to a certain extent from the higher perceptibility of [r]. In addition, and along the same lines, they may have been hindered in the recognition of words with the uvular fricative [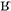] because of its higher perceptual confusability with other German back fricatives like the glottal fricative /h/. If any of these possibilities were true, this could have obscured the advantage that was expected to be found for [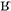] for this group.

## Conclusion

The present study provided a first assessment of how free allophonic variation of the German rhotic influences spoken word recognition for native and non-native listeners, while taking into account the status of the different variants in both L1 and L2, the perceptual properties of said variants and the characteristics of the phonological systems of the languages in question in a more general sense. Our results suggest that, for native German listeners residing in the south of Germany, the recognition of German words containing an initial /r/ is facilitated when it is produced as [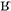], the allophonic variant that is considered to be canonical in standard varieties of German. Importantly, we show that canonicity has a substantive impact on word recognition even when confounding factors present in previous literature [e.g., orthographic (mis)matches] are accounted for and the sheer perceptual salience of the variants works against this outcome. For non-native listeners, by contrast, we observe patterns that are better explained by the influence of the L1 in terms of how /r/ is canonically produced. The status of the different variants in the L2 itself appear only secondary. Future research may build on these results by assessing issues such as individual variability within listener and learner groups, taking into account, for instance, how variant canonicity at a national level interacts with frequency of use in the particular environment of the individual listener.

## Data Availability Statement

The datasets presented in this study can be found in online repositories. The names of the repository/repositories and accession number(s) can be found at: https://osf.io/hn2e9/?view_only=8f6701fb4e4b4e36a82965066e451039.

## Ethics Statement

Ethical review and approval was not required for the study on human participants in accordance with the local legislation and institutional requirements. The patients/participants provided their written informed consent to participate in this study.

## Author Contributions

ER, NE, and ML designed the study. ER and NE implemented and ran the study. ML and ER analyzed the results and wrote the manuscript. All authors contributed to the article and approved of the final version.

## Conflict of Interest

The authors declare that the research was conducted in the absence of any commercial or financial relationships that could be construed as a potential conflict of interest.

## Publisher’s Note

All claims expressed in this article are solely those of the authors and do not necessarily represent those of their affiliated organizations, or those of the publisher, the editors and the reviewers. Any product that may be evaluated in this article, or claim that may be made by its manufacturer, is not guaranteed or endorsed by the publisher.

## Footnotes

1 We use /r/ to refer to the German rhotic phoneme as is common in the phonological literature on this topic (e.g., Wiese, 1996, 2003).

2 Note that some of these dialects of Italian tend to use rhotics other than the canonical alveolar trill. However, as the present question with regard to the L2 learner groups was about the possible influence of their L1 canonical rhotic on L2 word recognition, any experience learners potentially had with other rhotic allophones in their regional varieties would work against the hypothesis we outlined in section “The Present Study: Summary and Predictions.”

3 All three groups of participants show some variability in terms of the age and regional origin of participants, and Italian and French participants additionally span a range of age of learning German and (self-rated) proficiencies. While these factors are likely to affect each individual’s efficiency in German spoken word recognition in general and potentially also the recognition of the rhotic allophones examined, our goal was to compare the groups based on the canonical form of the rhotic in their L1. Once the overall pattern for these groups is unraveled in the present study, future studies using large sample sizes may start exploring more subtle patterns based on individual variation along all the various dimensions.

4 This overall effect of fewer target fixations by the learner groups relative to the native German listeners was replicated in an analysis of filler trials [*b*_(intercept)_ = 1.08, *t* = 5.34, *p* < 0.001, *b*_(French)_ = −1.18, *t* = −4.32, *p* < 0.001, *b*_(Italian)_ = −1.13, *t* = −3.82, *p* < 0.001]. This difference between native and non-native listeners was fully expected, since previous studies using similar methods and comparing similar populations have consistently reported considerable delays in word recognition for L2 learners (Weber and Cutler, 2004; Eger et al., 2019) compared to L1 listeners.
