# Supplementary material for: Free Allophonic Variation in Native and Second Language Spoken Word Recognition: The Case of the German Rhotic
Source: Front Psychol. 2021 Nov 17;12:711230. doi: 10.3389/fpsyg.2021.711230 (PMC8637905; doi:10.3389/fpsyg.2021.711230)
Supplement: Supplementary file 1 [file Data_Sheet_1.PDF]

Supplementary Material

Figure A1. Fixation proportions on critical trials with rhotic-initial targets separately for the three listener groups. They include not only target fixations, which are the focus of the analyses (see Figure 1 in the text), but also show fixations on competitors and distractors. Competitors were semantically related. Distractors were unrelated and mostly did not fit the sentence context, which explains the lower number of fixations in distractors already at target onset. The top panel shows fixations over time for native speakers of German (zero is acoustic target-onset), the middle panel shows data for the French learners and the bottom panel for the Italian learners. Dark lines represent trials in which targets were produced with the uvular fricative, lighter lines represent trials with targets produced with the alveolar trill. Target fixations are presented as solid lines, competitor fixations as dashed lines and distractor fixations as dotted lines. The grey vertical lines indicate the time-window of analyses.

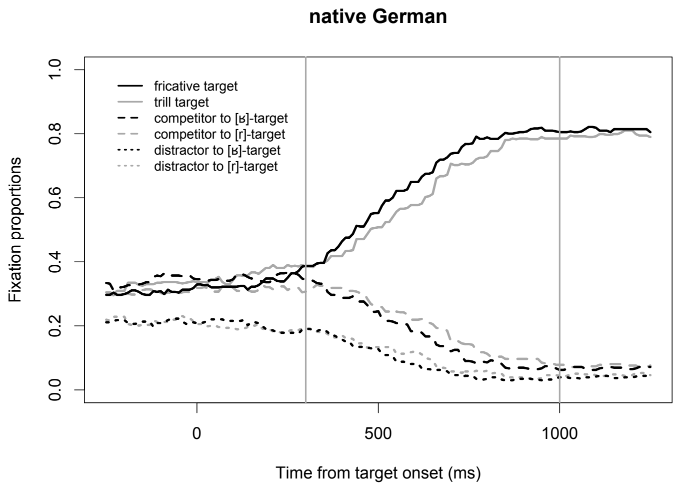

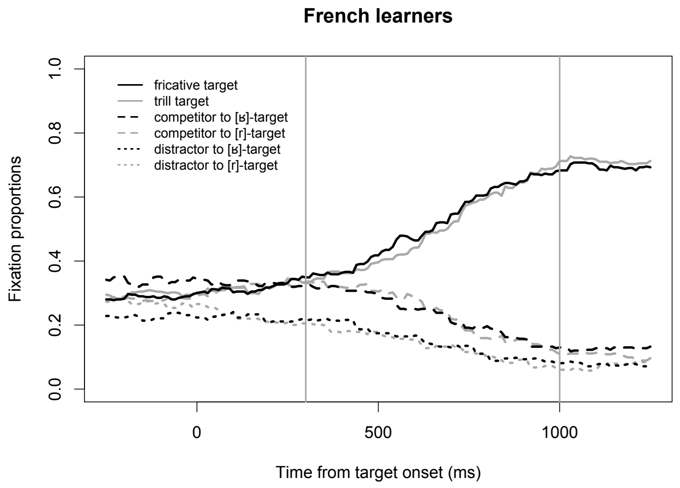

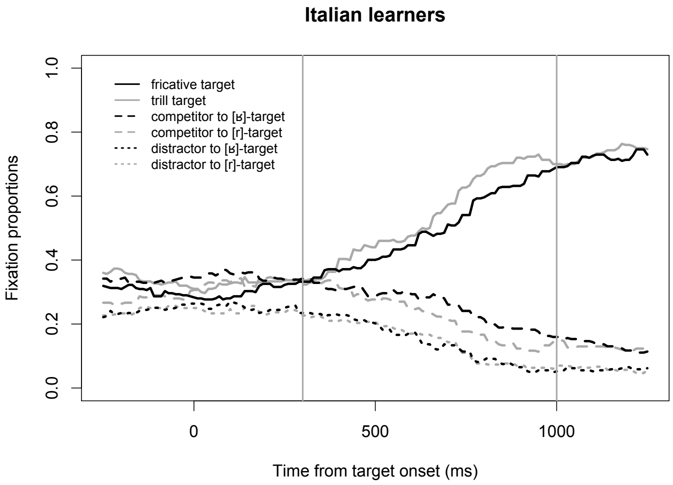
Italian learners
fricative target
trill target
competitor to [ʁ]-target
competitor to [r]-target
distractor to [ʁ]-target
distractor to [r]-target
Fixation proportions
0.0
0.2
0.4
0.6
0.8
1.0
0
500
1000
Time from target onset (ms)
